# An Algorithm for Modeling Thermoplastic Spherulite Growth Using Crystallization Kinetics

**DOI:** 10.3390/ma17143411

**Published:** 2024-07-10

**Authors:** Jamal F. Husseini, Evan J. Pineda, Scott E. Stapleton

**Affiliations:** 1 Department of Mechanical Engineering, University of Massachusetts Lowell, Lowell, MA 01854, USA; jamal_husseini@student.uml.edu (J.F.H.); scott_stapleton@uml.edu (S.E.S.); 2 Multiscale and Multiphysics Modeling Branch, NASA Glenn Research Center, Cleveland, OH 44135, USA

**Keywords:** thermoplastics, crystallization, modeling, spherulites

## Abstract

Crystallization kinetics were used to develop a spherulite growth model, which can determine local crystalline distributions through an optimization algorithm. Kinetics were used to simulate spherulite homogeneous nucleation, growth, and heterogeneous nucleation in a domain discretized into voxels. From this, an overall crystallinity was found, and an algorithm was used to find crystallinities of individual spherulites based on volume. Then, local crystallinities within the spherulites were found based on distance relative to the nucleus. Results show validation of this model to differential scanning calorimetry data for polyether ether ketone at different cooldown rates, and to experimental microscopic images of spherulite morphologies. Application of this model to various cooldown rates and the effect on crystalline distributions are also shown. This model serves as a tool for predicting the resulting semi-crystalline microstructures of polymers for different manufacturing methods. These can then be directly converted into a multiscale thermomechanical model.

## 1. Introduction

High performance semi-crystalline thermoplastic resins such as polyether-ether-ketone (PEEK) and polyether-ketone-ketone (PEKK) are gaining popularity for use in aerospace applications due to desirable material properties, manufacturability, and repairability while in service. The thermal history during manufacturing ultimately influences the growth and formation of microscale spherulites, which may impact the thermomechanical properties of these materials. For thermoplastics to be used for novel applications, understanding the relationship between the microscale crystalline morphology and bulk properties is imperative. Using this understanding, computational models can be developed that predict thermomechanical properties and residual stresses based on the material microstructure and spherulite growth derived from processing conditions.

At the microscale, semicrystalline thermoplastics contain structures referred to as spherulites, which consist of crystalline and amorphous phases with distinct mechanical properties. During thermal cooldown, spherulites grow radially from a nucleation site until they impinge on neighboring spherulites. As such, the material is not fully crystalline and there are gradients in the crystallinity of the spherulites themselves, resulting in a higher crystallinity at the core than the perimeter of the spherulites. Thus, the different spherulite morphologies and local crystalline distributions have an impact on the thermoelastic properties, residual stresses, and failure. 

Multiple studies have tried to examine spherulite fracture, but there is still not a complete consensus on this phenomenon [1,2,3]. Studies such as [1] have shown scanning electron microscope (SEM) images of a fracture surface where there is nuclei “pull-out” within the spherulite, whereas studies such as [2] indicate that cracks tend to propagate through the spherulite nucleus depending on its orientation. These studies highlight that understanding the crystalline microstructure of these materials is necessary to predict mechanical properties and failure. Understanding these phenomena is often difficult using experiments alone, as different manufacturing techniques such as ultrasonic welding, additive manufacturing, automated fiber placement (for composites), and stamp forming may produce unknown microstructures [4,5,6,7,8]. Computational simulation techniques can be used to establish structure–property relationships for thermoplastic materials and supplement experimental data.

Computational models have been developed to predict spherulite nucleation, growth, and crystallinity based on thermal history. These models typically use the Avrami equation [9] to model crystallization, which has shown good empirical agreement [10,11,12,13]. More modern approaches utilize crystallization kinetics, due originally to Lauritzen and Hoffman [14], to model crystalline growth [15]. From these methods, models have been developed which can describe the isothermal and non-isothermal crystallization of thermoplastic polymers [16,17,18,19,20,21,22]. Choe and Lee [10] reported an approach adopted from [12] to model the non-isothermal crystallization of PEEK. Their results showed that for different cooldown rates, the proposed model predicted the non-isothermal crystallization well as compared to experimental differential scanning calorimetry (DSC) values. Guan and Pitchuami [23] adapted this model to predict 2D PEEK spherulite growth around fibers based on processing parameters of a tow placement process. Saber [24] used crystallization kinetics to model spherulite growth in 2D and 3D domains with neat resin and with fibers, using scans of microstructures for fiber locations. These models were validated against cross-polarized microscopy images of in situ spherulite growth and relative crystallinity as a function of time in neat resin from DSC data, and then used in more complex model geometries. While these models serve as important tools in modeling spherulite growth, they do not address the distribution of crystalline and amorphous phases locally within the spherulites. If the ultimate goal is a thermomechanical model for predicting properties and failure, this additional level of detail is necessary. 

In this study, a crystallization kinetic model was developed from [10] using constants for PEEK and used to simulate different spherulite morphologies. Ultimately, the intent of the current development is to couple the output of the crystallization simulations to a multiscale thermomechanical model [25,26,27]. Therefore, the model domain was discretized into voxels, where, after the spherulite growth, each voxel was assigned crystallinity through an optimization scheme that used the overall crystallinity of the polymer as the objective. This optimization algorithm was used to determine the crystallinities of each spherulite and the local crystalline distributions within a spherulite. Results of this model were first validated against DSC data from the literature, as well as microscopic images of spherulites taken under cross-polarized light from [5]. Finally, different cooldown rates were simulated using this algorithm and the predictions presented. Coupling with multiscale thermomechanical models will be presented in subsequent publications. 

## 2. Materials and Methods

### 2.1. Spherulite Crystallization Growth Simulation

**Figure 1:**
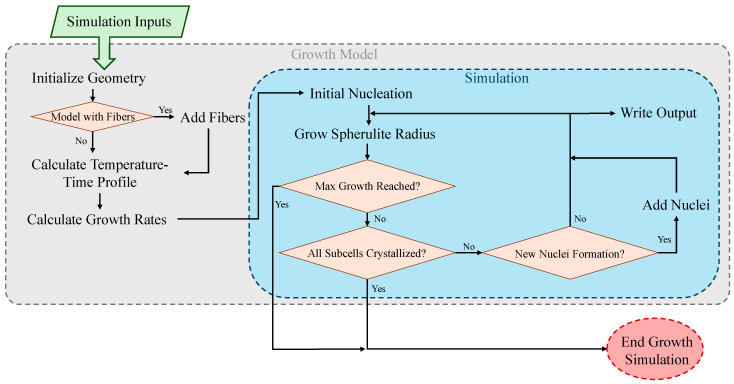
Flow chart describing process of thermoplastic spherulite growth simulation.

A simulation was developed that can model the growth of thermoplastic spherulites based on non-isothermal cooldown. The governing kinetic equations were introduced in [10] and applied in simulations by [23] for non-isothermal crystallization of PEEK. A flow chart showing the growth simulation process can be found in Figure 1.

The simulation works by discretizing a volume with lengths $l_{X}\times l_{Y}\times l_{Z}$ (or $l_{X}\times l_{Y}\;$ for a 2D simulation) into a grid of sub-volume elements (voxels) referred to as subcells for this study. The subcell length, which controls the coarseness of the subcell discretization, is defined by user input as $\;l_{s}$. The number of subcells in each coordinate direction is $l_{X}/l_{s}$, $l_{Y}/l_{s}$, and $l_{Z}/l_{s}$, respectively. The growth simulation assumes that the thermoplastic starts from a complete melt with no “memory” of previous nucleation, meaning that all nucleation events are random and not dependent on time history. This simulation also assumes that the entire volume considered is heated uniformly at each time increment. Once all input parameters defined by the user are preprocessed, the voxelated volume consisting of the subcells is constructed. If fibers are present in the simulation, the centers are placed on the x−y plane and are assumed to continue straight in the z-direction. Subcells within the fiber radius are identified and designated as regions where crystallization cannot occur. At t=0 s of the simulation, an instantaneous nucleation occurs and is governed by

(1) $$N=\frac{k_{1}}{4\pi v_{o}^{3}}$$

where N is the number of nuclei per unit volume, and $k_{1}$ and $v_{o}$ are kinetic constants for PEEK and can be found in Table 1. If there were fibers present, a fiber nucleation factor, $N_{F}$, was used and from the literature was assumed to be $N_{F}=4$ $\mathrm{nuclei}/{\mu m}^{2}$. $N_{F}$ only controls nucleation on the surface of the fiber. This is outlined to demonstrate that the algorithm can handle fiber surface nucleation, but henceforth this study will only present results without fibers. As the growth simulation continues, t>0 s, it was assumed that the spherulites are allowed to grow radially from each nucleation site. Note that the code could be easily altered to accommodate different growth patterns, such as parabolic growth from fiber surfaces, if deemed necessary. The growth rate of the spherulites, G, is described as

(2) $$G=v_{0}e^{\left(\frac{-E_{d}}{RT}\right)}e^{\left(\frac{-\psi_{1}T_{m}^{0}}{T\left(T_{m}^{0}-T\right)}\right)}$$

where $E_{d}$, $R,\psi_{1},$ and $T_{m}^{0}$ are kinetic parameters for PEEK outlined in Table 1. T is the current temperature based on the user prescribed cooldown rate and time step, highlighting that the simulation is both time- and temperature-dependent. When a subcell’s center was found to be within the growth radius of a spherulite nucleus, that subcell was “captured” as part of the spherulite. During the simulation, new nuclei were allowed to form based on the relation

(3) $$I_{nuc}=\frac{k_{2}}{4\pi v_{0}^{3}}e^{\left(\frac{-E_{d}}{RT}\right)}e^{\left(\frac{-\psi_{2}T_{m}^{0}}{T\left(T_{m}^{0}-T\right)}\right)}$$

where the kinetic constants $k_{2}$ and $\psi_{2}$ are also outlined in Table 1. $I_{nuc}$ is in units of $\left[\mathrm{Nuclei}/{\mu m}^{3}/s\right]$, where, for a given time increment, the nuclei per unit volume or $\left[\mathrm{Nuclei}/{\mu m}^{3}\right]$ is found and multiplied by the volume of amorphous subcells, or subcells which have not been captured by spherulite growth. If the number of nuclei is above one for that time increment, a new nucleus is added randomly to the domain. 

The growth simulation continues until the end of the cooldown or until there are no longer any subcells to capture within a spherulite. Periodicity was also enforced, where all fibers and spherulite growths were properly reflected over the simulation domain boundaries. 

### 2.2. Local Crystallinity Distribution

A spherulite is composed of lamella stacks that grow radially outwards from the nucleation center during the non-isothermal cool-down from melt [28,29]. Amorphous material occupies the volume in between crystalline lamella stacks. Due to the branch-like nature of lamella growing away from the nucleus, there is a local distribution of crystallinity within the spherulites that is important to capture in computational models when investigating the mechanical behavior of these materials [2]. 

From the growth simulation, relative crystallinity was calculated as the ratio of the number of subcells that were enveloped during the radial growth of the spherulites versus the total number of subcells. The relative crystallinity varies from zero, a pure amorphous material, to one, a material where spherulites have reached a maximum growth and occupy 100% of the volume of the material. 

For PEEK, a sample with a relative crystallinity of one may still only have an overall degree of crystallinity, χ, of 20–30% due to the inter-amorphous regions with spherulites between lamellae. To model this, each subcell within a spherulite was assigned a local crystallinity, $v_{c}$. This local crystallinity was optimized with the constraint that the crystallinity must decrease radially from the respective nucleus, and the objective function yielded the assumed maximum PEEK χ of 30% [30]. To this regard, if there were still uncaptured subcells once the simulation ended, χ was found by multiplying the relative crystallinity by the assumed maximum crystallinity, $\chi_{max}$, of 30%.

The optimization process started by calculating an overall degree of crystallinity, χ, as

(4) $$\chi =\chi_{V}\times \chi_{max}$$

where $\chi_{V}$ is the relative degree of crystallinity defined by

(5) $$\chi_{V}=\frac{V_{sph}}{V}$$

and V is the total volume of the domain, $V_{sph}$ is the volume of spherulites, and $\;\chi_{max}$ is the assumed maximum degree of crystallinity. 

Once χ was calculated, the crystallinity within each spherulite was found. It was assumed that smaller spherulites had a higher crystallinity than larger ones due to denser crystallization near the nucleus, which also composes more of the volume in small spherulites as compared to large ones. The crystallinity of each spherulite was found through minimizing an objective function

(6) $$\min|\chi_{sph}\cdot v_{sph}-\chi_{V}|$$

where $\chi_{sph}$ is a vector containing the crystallinities of individual spherulites ordered from lowest to highest and $v_{sph}$ is a vector containing the associated volumes. The optimization enforced a linear inequality such that

(7) $$A\;\chi_{sph}\leq b$$

where **A** and **b** are defined as

(8) $$A=\left[\begin{array}{cccc} -1 & 1 & 0 & \ldots \\ 0 & -1 & 1 & \ldots \\ 0 & 0 & -1 & 1\\ \ldots & \ldots & \ldots & -1 \end{array}\right]\;\;\mathrm{and}\;b=\left[\begin{array}{c} 0\\ 0\\ \ldots \end{array}\right].$$

The optimization was used to find $\;\chi_{sph}$, which achieved a target χ.

**Figure 2:**
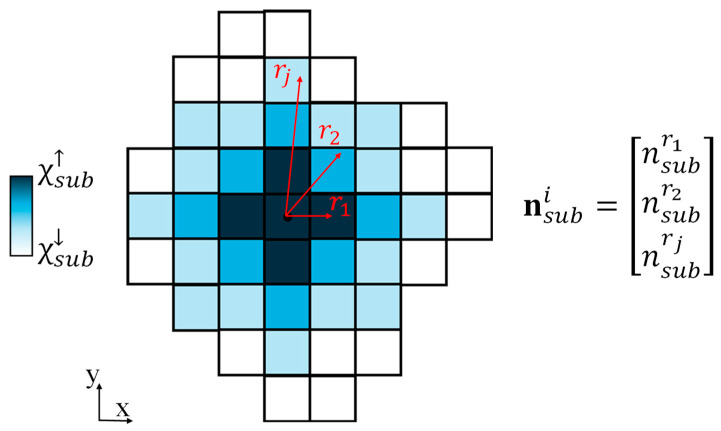
2D Representation of how crystallinity decreases radially from the spherulite nucleus where $n_{sub}^{i}$ is a vector containing the number of subcells assigned a specific crystallinity.

After the crystallinities of each spherulite were found in $\;\chi_{sph}$, the crystallinities of each subcell within a spherulite were determined using the same optimization process with a different objective function and linear inequality. For each spherulite i, the objective function is defined by

(9) $$\min\left|\frac{\chi_{sub}\cdot n_{sub}^{i}}{\sum n_{sub}^{i}}-\chi_{sph}^{i}\right|$$

where $\chi_{sub}$ is a vector containing the crystallinities of subcells within a spherulite, $n_{sub}^{i}$ is a vector containing the number of subcells with centroids located at particular radial distances from the nucleus where the *j*th entry of$\;n_{sub}^{i}$ is the number of subcells within a radius, $r_{j}$. A schematic of this is shown in Figure 2 depicting how $n_{sub}^{i}$ is determined for an arbitrary spherulite demonstrated in a 2D cross section. The subcells that fall within a given radius are all assigned the same crystallinity determined from the optimization. The summation of $n_{sub}^{i}$ is the total number of subcells within a spherulite. It is assumed that the subcells at the nucleus have a fixed crystallinity of 85%, which is approximately the maximum possible crystallinity [26]. The linear inequality for this optimization is defined as

(10) $$C\;\chi_{sub}\leq d$$

where **C** and **d** are defined as

(11) $$C=\left[\begin{array}{cccc} -1 & 1 & 0 & \ldots \\ 0 & -1 & 1 & \ldots \\ 0 & 0 & -1 & 1\\ \ldots & \ldots & \ldots & -1 \end{array}\right]\;\;\mathrm{and}\;d=\left[\begin{array}{c} 0\\ 0\\ \ldots \end{array}\right].$$

## 3. Results and Discussion

### 3.1. Parameter Sensitivity and Validation

**Figure 3:**
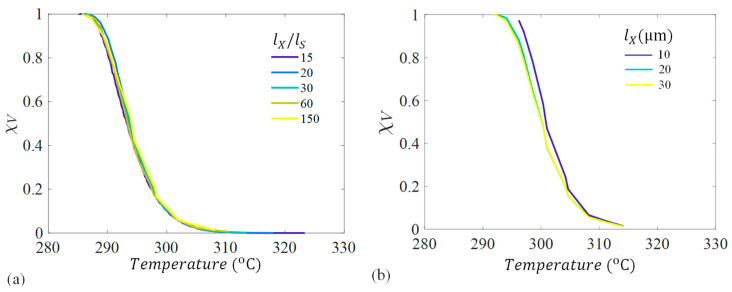
Relative crystallinity, $\chi_{V}$, versus temperature for a simulation with a 10 °C/min cooldown for (**a**) increasing subcell refinement and (**b**) increasing domain size.

A parameter convergence study was conducted to determine the maximum subcell refinement required to capture the crystallinity evolution accurately. A cube domain was used where all lengths were equal (i.e., $l_{X}=l_{Y}=l_{Z})$, the subcell dimension, $l_{s}$, was varied, and the crystallinity outputs were measured. The domain size was normalized by subcell length $\left(\frac{\;l_{X}}{l_{s}}\right)$, where the minimum subcell length could be determined based on the desired domain. To examine this, a thermal cooldown of 10 °C/min was applied to a domain where $l_{X}=15\;\mu m$ and the subcell length was refined from 1 μm to 0.1 μm and the relative crystallinity, $\chi_{V}$, was recorded. The domain size was also varied from 10 μm to 30 μm keeping $l_{s}=1$ constant. The results are shown in Figure 3.

Shown in Figure 3a, there is minimal dependency on subcell refinement for the crystal growth, but Figure 3b shows that domain size does affect the spherulite volume fraction. This domain size dependency is likely due to homogeneous nucleation because a larger overall volume means that more nuclei can form during cooldown. This is why the curves start at the same temperature at $\chi_{V}=0$, but the new nuclei that form in the larger domains cause the curves to differ from smaller domains. For the remainder of the results, a standard domain size of 30 μm and a subcell refinement of 1 μm were used. 

**Figure 4:**
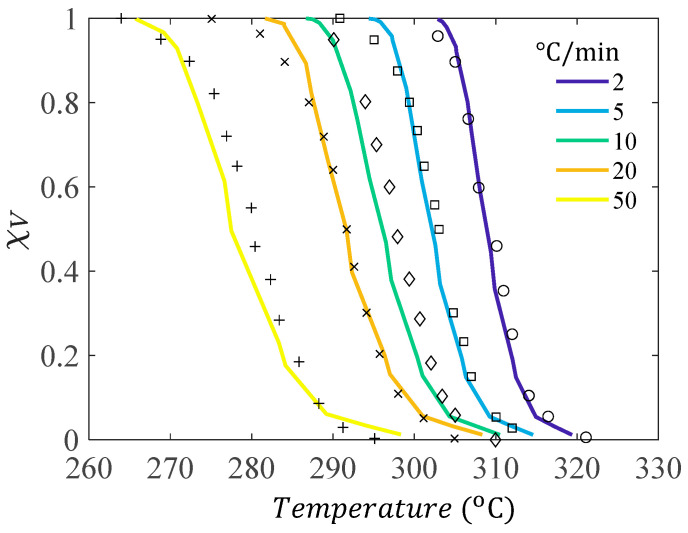
Validation of crystallization kinetic model with experimental DSC results [10] for thermal cooldowns of 2(+), 5(×), 10(◊), 20(□), and 50(o) °C/min.

A validation of the crystallization kinetic mode was performed, where different non-isothermal cooldown rates were applied and $\chi_{V}$ was recorded. The model predictions were compared to differential scanning calorimetry (DSC) experiments from the literature for PEEK [10]. Figure 4 shows that there is very good agreement between the experimental and simulation results, which implies that the kinetic constants used (Table 1) can predict the spherulite growth for PEEK for different non-isothermal cooldown rates. There are slight differences at high $\chi_{V}$, which is possibly due to the domain being discretized into voxels, causing jumps in $\chi_{V}$ when a new subcell is captured, or nucleates. 

.

**Figure 5:**
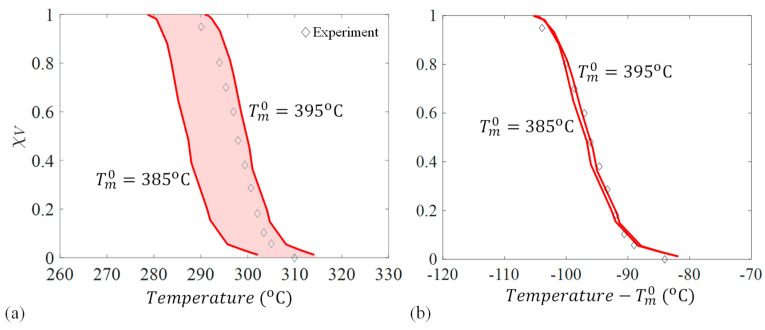
(**a**) Sensitivity of relative crystallinity, $\chi_{V}$, based on reported equilibrium melting temperatures, $T_{m}^{0}$, and (**b**) adjusted with respect to $T_{m}^{0}$ for PEEK.

It was noted in this study that changing equilibrium temperature,$\;T_{m}^{0}$, which for PEEK is commonly reported as 395 °C [31] but was measured in [10] as 385 °C, resulted in a shift of relative crystallinity. The effect of these two bounds on recorded $\chi_{V}$ was compared to the experimental results [10] at a 10 °C/min cooldown and shown in Figure 5a. Figure 5a shows that $\chi_{V}$ shifts by approximately 10 °C when $T_{m}^{0}$ changed, and this value had to be chosen carefully to represent experimental results. The shaded area in between the two bounds shows where results may fall if an intermediate $T_{m}^{0}$ value is chosen. For this study, a $T_{m}^{0}=392\;{}^{\circ}C$ was chosen for thermal cooldowns 10 °C/min and below. By contrast, for higher thermal cooldowns, $T_{m}^{0}=395\;{}^{\circ}C$ was used, which better represented the experimental results. This shift in relative crystallinity, which is almost equal to the difference in $T_{m}^{0}\;$ bounds, suggests that relative crystallinity is nearly constant with respect to the temperature to the equilibrium melting point, shown in Figure 5b. Figure 5b shows that the curves line up closely when adjusted based on respective $T_{m}^{0}$. Moreover, this aspect can be beneficial for future simulations with new materials where $T_{m}^{0}$ may not be known.

### 3.2. Spherulite Morphology Validation

**Figure 6:**
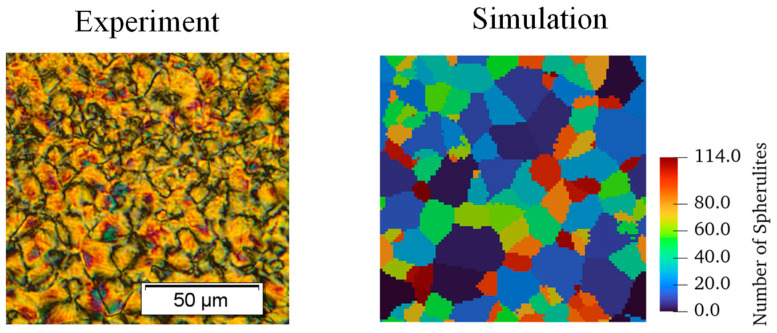
Spherulite morphologic comparison between a thin PEEK sample cooled at 20 °C/min [5] and simulation.

Once the model was validated to ensure the spherulite growth was representative of experimental DSC results, it was used to compare simulated morphologies to experimental scans. A study by [5] was simulated where a film of PEEK was cooled at 20 °C/min. For the simulation, a domain size of 100×100×1 μm was used with a subcell length of 1 μm. A side-by-side comparison is shown in Figure 6.

**Figure 7:**
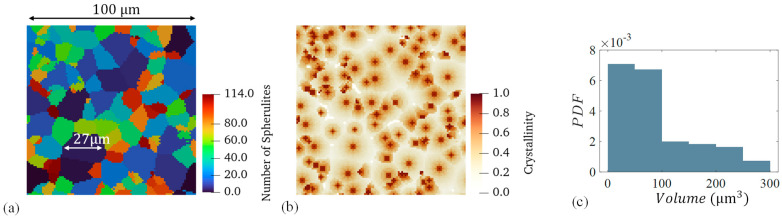
(**a**) Spherulite morphology from a sample cooled at 20 °C/min with the largest spherulite diameter labelled, (**b**) the corresponding local crystallinity distribution, and (**c**) a probability density function (PDF) of spherulite volumes.

The results reported by [5] showed that there was a distribution in spherulite sizes of approximately 15–30 μm. The simulation shows similar results with spherulite measurements depicted in Figure 7.

In Figure 7a, the growth simulation showed similar spherulite measurements compared to experiments where the largest spherulite had a diameter in the upper end of the reported range. Figure 7c shows the probability distribution function (PDF) of all spherulite volumes. Spherulites are not perfectly circular, and their volumes do not directly correspond to their diameter, which is typically reported for these experimental measurements. Figure 7b shows the calculated crystallinity distribution for this sample, where the nuclei are clearly visible. The results show the simulation is producing expected spherulite sizes compared to experimental results. More advanced image analysis techniques are needed to measure and validate the crystallinity distribution within spherulites in these types of experimental results.

**Figure 8:**
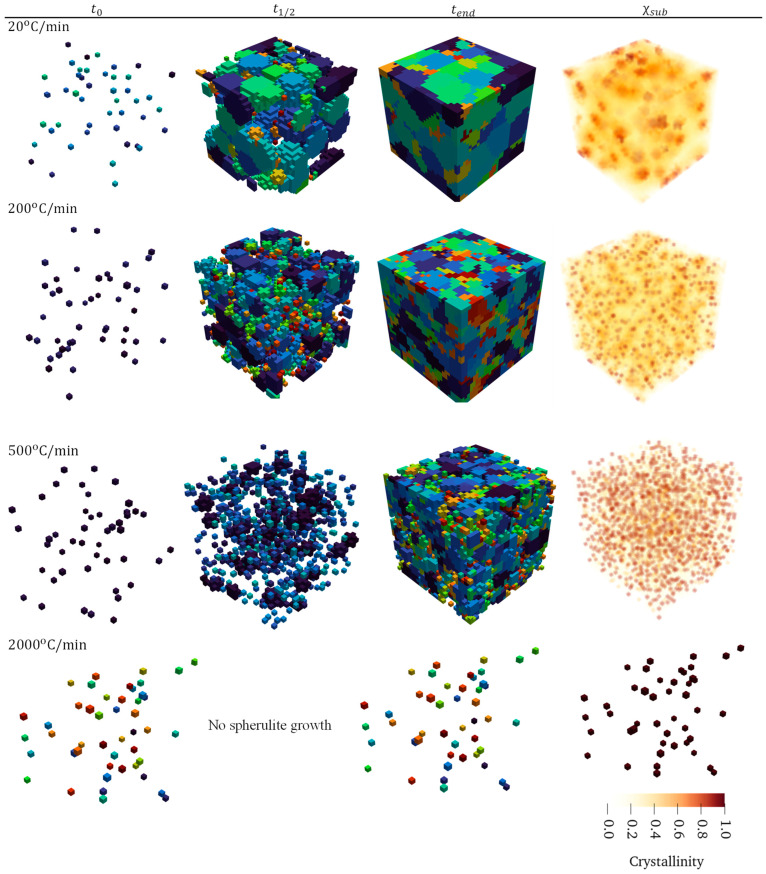
Spherulite nucleation, growth, and crystallinity for cooldown rates of 20, 200, 500, and 2000 °C/min compared at the initial time step, halfway through the simulation, and the final time step, along with the final subcell crystallinity distribution. The spherulite colors in the first three columns represent spherulite numbers.

Typical cooldown rates may vary depending on manufacturing and application. For example, tow placement processes such as automated fiber placement can have a cooldown rate on the order of 2000 °C/min [32]. To examine this effect on spherulite growth and morphology, simulations with different cooldown rates (20, 200, 500 and 2000 °C/min) were conducted using a 30×30×30 μm domain size and compared in Figure 8.

**Figure 9:**
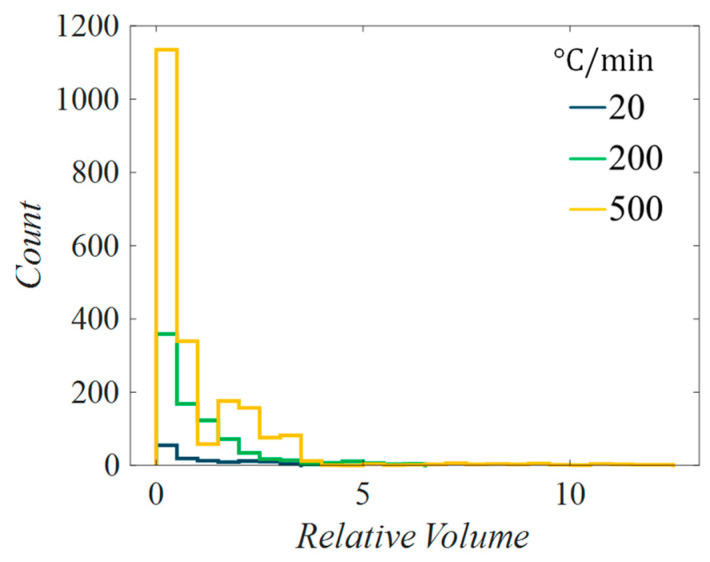
Number of spherulites versus relative volume for simulations of three different cooldown rates.

**Figure 10:**
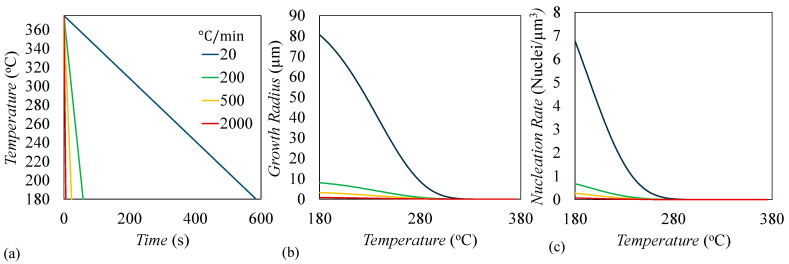
(**a**) Temperature versus time, (**b**) nuclei growth radius, and (**c**) nucleation rate for four different simulations with increasing thermal cooldown rates.

Figure 8 shows the nucleation, spherulite evolution, and resulting local crystallinity for different cooldown rates. Figures of the growth were taken at initial nucleation, halfway through the cool down, and at the final time step. Both 20 and 200 °C/min reached a state with fully grown spherulites that impinged with one another, where the cooldown of 500 °C/min had some spherulite growth but not full impingement and 2000 °C/min was quick enough to not let the spherulites grow past initial nucleation or form new nuclei. As a result, the crystallinity of each nucleus in the 2000 °C/min simulation was equal to 85% where the surrounding is pure amorphous. It can also be seen that the 20 °C/min simulation allowed for spherulites to grow significantly larger than the 200 °C/min simulation, but more new nuclei formed in the latter case. The result shows that a slower cooldown rate promotes spherulite growth, whereas a higher cooldown rate promotes more nucleation with smaller spherulites, which is consistent with experimental results shown in [33,34]. This relationship between growth and cooldown rates can be seen in Table 2, except for 2000 °C/min, because there was no spherulite growth. A distribution of spherulite relative volumes, the volume of each spherulite divided by its respective average, can be seen in Figure 9. The crystallinity in Figure 8 also represents this finding and shows that larger spherulites have a gradual crystalline transition from the nucleus outwards. The relationship between thermal cooldown, growth radius, and homogeneous nucleation rate for these cases is shown in Figure 10.

Table 2 shows that the minimum spherulite volume for the 200 °C/min and 500 °C/min cooldown was $1\;{\mu m}^{3}$, meaning it was only a nucleus that did not grow because the subcell dimension was $l_{s}=1\times 1\times 1\;\mu m$. This can occur when a new nucleus is placed in between surrounding spherulites and is not given any room to grow, or near the end of a fast cooldown where growth rate is very low, and the final temperature is reached before growth to a neighboring subcell is reached. This can be changed by increasing the subcell refinement, which will decrease the probability that a nucleus will be fully surrounded by spherulites. Further, lower growth rates could be captured because the subcell-to-subcell distance would be smaller. While this may be desirable, increasing the discretization will increase computation time, and further studies are needed to understand required refinement to achieve sufficient fidelity. The 20 °C/min simulation has the tightest distribution where bins are similar heights, meaning the spherulites are similar volumes. As the cooldown rate increases, so does the number of small spherulites, as well as the distribution spread, meaning that there is a large range of spherulite sizes relative to the average.

**Figure 11:**
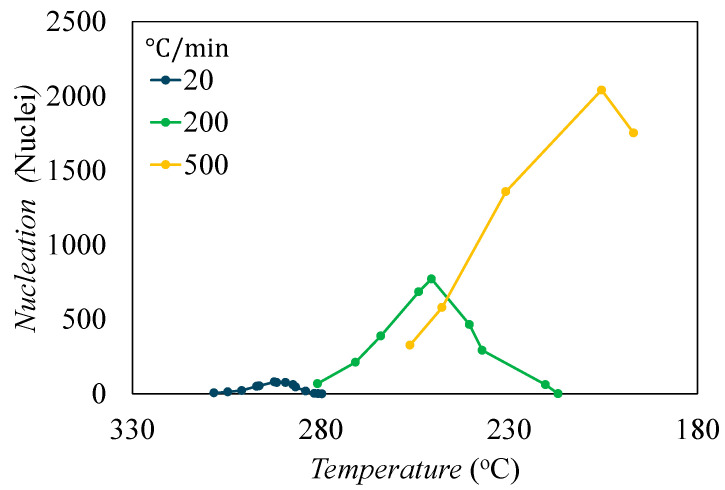
Nucleation versus temperature for three different thermal cooldowns.

Figure 10a shows the temperature versus time profile for the four examined cooldown rates. Figure 10b shows the corresponding growth radius for all cooldown rates, which is calculated by integrating Equation (2) with respect to time. This shows that the slower the thermal cooldown, the larger the growth radius can form by the end of the simulation. Lastly, Figure 10c shows the homogeneous nucleation rate as a function of temperature, which is calculated by integrating Equation (3) with respect to time and shows similar relationships of the growth radius. While this shows that slower cooldowns should form more nuclei per unit volume, this is opposite of what is displayed in Figure 7. This is because as the growth radius increases, the amount of subcells not captured by a spherulite decreases, so in slower cooldowns, there is significantly less volume for new nucleation. By contrast, in fast cooldowns, the growth radius is not very large and there is a large amount of remaining amorphous volume, so when the nucleation rate is multiplied by the available amorphous volume to determine how many nuclei form, that value is higher than slower cooldowns shown in Figure 11.

Figure 11 shows the number of nuclei that form during the thermal cooldown rates. This is found by multiplying nucleation rate (Figure 10) by the amorphous volume at a given time step. Seen in Figure 11, nucleation happens at different temperature ranges for each of the cooldowns. At the temperature range of the 20 °C/min simulation, all three cooldown rates have very similar nucleation rates (Figure 10c), but the spherulite growth radius is much higher, meaning there is not much amorphous volume, resulting in low nuclei formation. This shows how the 20 °C/min cooldown has fewer, larger spherulites, and at ~280 °C there is full impingement. The 200 °C/min nucleation primarily happens in a temperature range of 280 °C–230 °C where the spherulite growth radius for this cooldown rate is low and has a higher nucleation rate than the 20 °C/min cooldown. Due to the spherulites not growing as large, there is more amorphous volume available, and more nucleation can occur. The 500 °C/min cooldown has the lowest spherulite growth radius of the three cases, and a lower nucleation rate than the 200 °C/min cooldown. But, because there is less growth, there is significantly more amorphous volume available, and the nucleation is much higher. The 2000 °C/min case had no new nucleation or growth.

## 4. Conclusions

In this study, a model was developed that uses crystallization kinetics to model the growth of semi-crystalline spherulites within a domain. Optimization was used, with assumed constraints on spherulite geometries, to determine inter-spherulitic crystallinities. The kinetic constants and equations used in this model were obtained from the previous literature for PEEK. The equations were applied to a model domain that was discretized into voxelated regions called subcells. After growing the spherulites in this domain, overall crystallinity was determined, and optimization schemes were used to determine crystallinities for each spherulite and then crystallinities for each subcell. Results of this study investigated the model validation against DSC data for PEEK from literature, and then sensitivity of the model to domain size, subcell discretization, and certain kinetic constants. Then, a simulated microstructure was compared to an experimental image from PEEK after a cooldown where spherulite sizes were compared. Finally, the effect of cooldown rate was examined by simulating three rates of 20, 200, 500 and 2000 °C/min. The results of these simulations showed that the fastest cooldown rate resulted in no spherulite growth, just nucleation, the rate of 20 °C/min showed fewer but larger spherulites grown, 200 °C/min showed more but smaller spherulites, and 500 °C/min showed some spherulite growth but not full impingement. These models were then processed through the optimization schemes and the final crystalline distributions were shown. The crystalline distributions show the nucleus of the spherulites having higher crystallinities that decrease radially outward. 

Previous studies have shown that thermomechanical modeling of thermoplastics is a multiscale problem due to the local distribution and orientation of the lamellae, and the current model was developed to directly convert into a geometry for NASA’s Multiscale Analysis Tool (NASMAT), which relies on subcell discretization for its semi-analytical multiscale recursive micromechanics methods [25,35,36,37]. Knowing the crystallinity for each individual subcell from this analysis allows for lower length scales, such as individual lamella surrounded by amorphous material, to be simulated and integrated into the mesoscale containing the morphology of the spherulites. Coupling with NASMAT will also allow for linear and nonlinear thermomechanical analysis, where stiffness, strength, fracture toughness, thermal conductivity, etc., can be simulated for different morphologies resulting from different manufacturing processes. Future work on this model will include studying the effect of these properties with the inclusion of fibers, where fiber morphologies [38] and different spherulite geometries from spherulite nucleation on the fiber surface can impact mechanical results. Finally, an anisotropic core for the spherulites will be considered as this impacts the fracture behavior [2]. 

## Figures and Tables

**Table 1:** List of PEEK crystallization kinetic values used as inputs for spherulite growth simulation.

Symbol	Value	Units
$v_{o}$	$7.50\times 10^{8}$	$\left[\mu m/s\right]$
R	1.986	[cal/molK]
$k_{1}$	$9.03\times 10^{24}$	$\left[s^{-3}\right]$
$k_{2}$	$9.32\times 10^{38}$	$\left[s^{-4}\right]$
$T_{m}^{0}$	385–395	$\left[{}^{\circ}C\right]$
$E_{d}$	$1.52\times 10^{4}$	$\left[\mathrm{cal}/\mathrm{mol}\right]$
$\psi_{1}$	529	$\left[{}^{\circ}C\right]$
$\psi_{2}$	1517	$\left[{}^{\circ}C\right]$

**Table 2:** Minimum, maximum, average, and standard deviation of spherulite volumes from three different thermal cooldowns.

Cooldown (°C/min)	Minimum Volume (μm^3^)	Maximum Volume (μm^3^)	Average (μm^3^)	Standard Deviation
20	4	767	221.31	212.91
200	1	213	32.88	35.61
500	1	106	8.60	12.81

## Data Availability

Dataset available on request from the authors.
